# Outcomes of a Citywide Campaign to Reduce Medicaid Hospital Readmissions With Connection to Primary Care Within 7 Days of Hospital Discharge

**DOI:** 10.1001/jamanetworkopen.2018.7369

**Published:** 2019-01-25

**Authors:** Dawn Wiest, Qiang Yang, Carter Wilson, Natasha Dravid

**Affiliations:** 1 Camden Coalition of Healthcare Providers, Camden, New Jersey

## Abstract

**Importance:**

Previous research suggests the important role of timely primary care follow-up in reducing hospital readmissions, although effectiveness varies by program design and patients’ readmission risk level.

**Objective:**

To evaluate the outcomes of the 7-Day Pledge program to reduce readmissions by increasing access to timely primary care appointments after hospitalization.

**Design, Setting, and Participants:**

Retrospective cohort study of hospital readmissions among Medicaid patients 18 years or older hospitalized from January 1, 2014, to April 30, 2016, in Camden, New Jersey. To assess each patient’s hospital use before and after hospital discharge, all-payer claims data from 4 health care systems were linked to insurers’ lists of patients assigned to Camden-based primary care practices. A total of 1531 records were categorized by timing of a primary care appointment after discharge. Discharges followed by a primary care appointment within 7 days (treatment group) were matched by propensity scores to those with less timely or no primary care follow-up (nontreatment pool).

**Interventions:**

Targeted patient enrollment during hospital admission, primary care practice engagement, patient incentives to overcome barriers to keeping an appointment, and reimbursements to practices for prioritizing patients recently discharged from the hospital.

**Main Outcomes and Measures:**

The primary outcome was the number of hospital discharges followed by a readmission within 30 days. The secondary outcome was the number of hospital discharges followed by a readmission within 90 days.

**Results:**

There were 2580 hospitalizations of patients 18 years and older included on the patient lists from January 1, 2014, to April 30, 2016. Of these, 1531 records categorized by timing of a primary care appointment after discharge were studied. The treatment group consisted of 450 discharged patients (mean [SD] age, 48.7 [14.7] years; 289 [64.2%] female; 203 [45.1%] black, non-Hispanic). The nontreatment pool consisted of 1081 discharged patients (mean [SD] age, 48.1 [14.9] years; 599 [55.4%] female; 526 [48.7%] black, non-Hispanic). Among this cohort, the number of discharges followed by any readmission was lower for patients with a primary care visit within 7 days of hospital discharge than for their matched referents at 30 days (57 of 450 [12.7%] vs 78.8 of 450 [17.5%]; *P* = .03) and 90 days (126 of 450 [28.0%] vs 174 of 450 [38.7%]; *P* = .002) after discharge.

**Conclusions and Relevance:**

Facilitated receipt of primary care follow-up within 7 days of hospital discharge was associated with fewer Medicaid readmissions. The findings illuminate the importance of reducing barriers that patients and providers face during care transitions.

## Key Points

**Question:**

Is connection to primary care within 7 days of hospital discharge associated with reduced readmissions?

**Findings:**

In this cohort study of 1531 hospital discharges among adult Medicaid patients in Camden, New Jersey, patients attending a primary care follow-up appointment as part of the 7-Day Pledge program had fewer 30- and 90-day readmissions compared with patients with less timely primary care follow-up or none.

**Meaning:**

The findings suggest that facilitated connection of patients to primary care within 7 days of hospital discharge is associated with fewer hospital readmissions.

## Teaser

This cohort study of adult Medicaid patients discharged from the hospital assesses the outcomes of a program to reduce readmissions by increasing access to timely primary care appointments after hospitalization.

## Introduction

Hospital readmissions are costly indicators of the quality of patient care.^1,2^ In 2012, 7% of the US population had a hospital stay, for a total cost of $381 billion,^3^ and an estimated 1 in 12 adults is readmitted within 30 days, adding $16 billion to health care costs annually.^4^ To address costs and improve care quality, the Patient Protection and Affordable Care Act established the Centers for Medicare & Medicaid Services’ Hospital Readmissions Reduction Program. Launched in October 2012, this program ties payments for all Medicare admissions to hospitals’ readmission rates, creating motivation for hospitals to reduce avoidable readmissions.^2^ An estimated one-quarter of all 30-day hospital readmissions are preventable, and many of these can be traced to inadequate postdischarge follow-up.^5^ Timely postdischarge follow-up with a primary care provider (ie, a physician, nurse practitioner, clinical nurse specialist, or physician assistant as allowed under state law) can improve care transitions and reduce readmission risk through medication reconciliation, discharge plan review, and patient education.^6,7^

However, whether a patient schedules and attends a primary care appointment after hospital discharge depends on various factors. Geographic accessibility, often correlated with race and economic status, matters.^8,9,10^ Wait time, inability to secure timely appointments, and practice hours can keep patients from scheduling or attending a follow-up appointment.^11^ Patients’ past experiences with health care providers influence whether, where, and when they seek care.^12^ A previous study^13^ also highlighted the effect of insurance status and physician reimbursement models: in 2015, approximately 69% of physicians nationally were accepting new Medicaid patients vs 89% new private insurance patients.

Efforts to reduce hospital readmissions through timely primary care follow-up produced mixed results. A randomized clinical trial of an intervention using electronic health record–generated reminders to primary care practices to schedule visits for older adult patients within 7 days of hospital discharge had no effect on primary care follow-up rates and did not reduce readmissions.^14^ In contrast, a randomized clinical trial of the Care Transitions Intervention found significantly reduced readmissions at 30 and 90 days for older adults who received timely postdischarge follow-up as part of an intensive care transition intervention.^6^ Similarly, an observational study^15^ of a hospital-to-home transitional care initiative that coordinated connection to a primary care medical home for Medicaid recipients found fewer readmissions among patients receiving the intervention compared with patients receiving usual care. Discrepant effects of programs on reducing readmissions may signal the importance of timely primary care follow-up as one factor in reducing readmission risk for designated patient populations.^16^

Other research^17,18^ found that the effectiveness of timely primary care follow-up in reducing readmissions depends on the target population’s level of medical and social complexity. A study^17^ of 65 085 North Carolina Medicaid discharges found that people with the highest risk benefited most from primary care follow-up within 7 days of discharge, whereas patients in the lowest risk categories gained little benefit from early follow-up. Studying the intersection of homelessness, mental illness, and health care utilization, researchers found that receiving outpatient services within 7 days of a hospital discharge did not reduce readmissions among homeless people struggling with mental illness, suggesting that postdischarge care may be necessary but not sufficient for reducing readmission risk for the most vulnerable populations.^18^

The objective of this study was to evaluate outcomes of a citywide campaign in Camden, New Jersey, to reduce readmissions by increasing access to timely primary care follow-up after a hospitalization. In Camden, where Medicaid covers 57% of residents^19^ compared with 20% nationally,^20^ service provision is fragmented and resources are limited. Run by the Camden Coalition of Healthcare Providers (Camden Coalition), the 7-Day Pledge (7-DP) program leveraged a citywide data infrastructure, 12 primary care practices, and a care team that engaged patients while they were still in the hospital to connect them to a primary care appointment within 7 days of a hospital discharge. The 7-DP program addressed multiple barriers to primary care access. For patients, predischarge engagement to discuss the benefits of primary care follow-up and schedule visits, transportation to and from the primary care office, and a $20 gift card after appointment completion helped reduce barriers. For practices, the program offered an enhanced reimbursement on top of regular reimbursement and value-based payments: $100 for visits within 14 days and $150 for visits within 7 days were given to cover the resources required to prioritize recently discharged patients. Camden Coalition staff met monthly with each practice to review the 7-DP data and discuss strategies for improving program implementation.

## Methods

### Data Sources and Setting

In this retrospective cohort study, we identified all hospitalizations for patients 18 years or older from January 1, 2014, to April 30, 2016, that were reviewed for 7-DP eligibility. Records for Medicaid patients hospitalized in Camden and assigned by insurers to a Camden-based primary care practice were reviewed. Pregnancy-related, labor and delivery, oncology-related, psychiatric only, and surgery-related admissions were ineligible for the program because a primary care visit was not considered to be the most appropriate follow-up care. Patients hospitalized after a motor vehicle crash were also ineligible because of administrative barriers related to car insurance coverage of follow-up visits for motor vehicle crashes. Patients discharged to a short- or long-term care facility after their hospital admission or who were enrolled in the Camden Coalition’s intensive care management program at the time of their hospitalization were also ineligible. However, those who had access to home health care or an Assertive Community Treatment program were eligible because in many cases these services do not replace a patient’s primary care provider. The Cooper University Hospital Institutional Review Board determined that this study was a quality improvement study and waived review. This study adhered to the Standards for Quality Improvement Reporting Excellence (SQUIRE) reporting guideline. Only patients who provided oral informed consent for the data to go into the health information exchange were included in the program and analysis. Data were not deidentified. Patient name, date of birth, sex, and Social Security number were included in these data, and these variables were used to link records across multiple data sources.

UnitedHealthcare and Horizon NJ Health provided the Camden Coalition with monthly lists of Medicaid patients assigned to Camden-based primary care offices. Lists were uploaded into the Camden Health Information Exchange (CHIE), where daily admit-discharge-transfer data from 4 regional health care systems were housed. Once these lists were linked to the CHIE, a daily report of all admissions from these individuals was generated and imported into the client tracking database. Using this list, which was reviewed daily for program eligibility, program staff aimed to meet patients at hospital bedside or to reach them by telephone if an in-person visit was impossible to schedule a primary care appointment within 7 days of estimated discharge. Appointments scheduled for patients who were still admitted on their appointment date were rescheduled. On completion of the visit, practice staff entered the visit date into the database. Camden Coalition staff followed up with practices monthly to check the status of patients for whom no information about a postdischarge appointment was entered, correcting information as necessary.

To assess each patient’s hospital utilization before participating in 7-DP, all-payer claims data from 4 regional health care systems were linked to patient lists using multistage deterministic linkage (eAppendix 1 in the Supplement). These data were also used to measure hospital utilization 30 and 90 days after the discharge connected to the hospitalization accounted for in the workflow (ie, the index discharge) and to validate the index hospitalizations flagged in the CHIE. We counted all hospital admissions within 30 and 90 days of a discharge as a readmission except those that would make the patient ineligible for the program. If a readmission occurred within 7 days of the index discharge and before a primary care appointment, we used that readmission as the index admission.

### Outcomes

The primary outcome was the number of index discharges followed by 1 or more hospitalizations within 30 days, a common follow-up period measured in the literature.^5,6,7,16,21,22,23^ A secondary outcome was the number of index discharges followed by 1 or more hospitalizations within 90 days.

### Statistical Analysis

Because primary care visits within 7 days of discharge were not randomly assigned, we used propensity score matching to reduce the imbalance of measured patient characteristics between the treatment group and nontreatment pool (eAppendix 2 in the Supplement). Each propensity score value is the probability that a hospital discharge would be followed by a primary care visit within 7 days conditional on measured covariates.

We fit a multivariate logistic regression model with the following covariates to estimate propensity score values: age, sex, race/ethnicity, physical illness severity, substance-related diagnoses, mental health–related diagnoses, and number of hospitalizations and emergency department visits in the 6-month period before the index admission. Race/ethnicity was coded from the hospital claims data and was included because of its association with primary care access.^8,9,10^ To determine the severity of physical illness, we used the Quan Index, an updated version of the Charlson Comorbidity Index, which uses *International Classification of Diseases* codes in claims data to categorize patients’ comorbidities.^24,25^ Each comorbidity category has an associated weight, ranging from 1 (least severe) to 6 (most severe), based on the adjusted risk of mortality or resource use. The sum of all the weights results in a single comorbidity score for a patient.

For each patient in the treatment group, we used nearest neighbor matching with replacement to identify 5 matched referents in the nontreatment pool.^26^ After finding the 5 nearest records based on an absolute difference of 0.01 or less between propensity scores, we took the mean to build a comparison record for each record in the treatment group. Our propensity score matching method resulted in a reasonable balance for each covariate based on a standardized bias of less than 0.1 (eTable 1 in the Supplement) and acceptable overlap between treatment and nontreatment propensity scores (eFigure in the Supplement).

Our propensity score matching method only selected a portion of comparison records. Therefore, we also reweighted the records by assigning weights to each record in the nontreatment group based on their propensity scores and reran the analysis. Nontreatment records that were similar to treatment records were given higher weights, whereas nontreatment records that were dissimilar to treatment records were given smaller weights. The weight for each record *i* in the nontreatment group was *p*_i_/(1− *p*_i_), where *p_i_* was the record’s propensity score. We then rescaled the weights within the treatment and nontreatment groups so the proportions after reweighting were similar to those observed empirically between the 2 groups.

Subsequent sensitivity analyses examined readmission outcomes with alternative models. First, to allow time for treatment exposure, we shifted the timing for counting readmissions from day 1 to day 8 after discharge for all records. Second, we recalculated the propensity scores after removing all records for which the patient declined the help of program staff in scheduling an appointment (n = 52) or who were unresponsive to staff attempts to engage them during the index admission or shortly after discharge when staff attempted to reach them by telephone or a letter sent to their home (n = 219).

To test whether there was an association between timing of primary care follow-up and readmissions, we used 2-sample z tests with modified standard errors (eAppendix 3 in the Supplement) to compare outcome variables by treatment status. In bivariate analyses, 2-sample z tests were used to test for significant differences in treatment exposure among subgroups of patients. A log-likelihood test was used to determine the probability of treatment as a function of all covariates in the logistic regression model used to generate propensity scores. The significance level for the primary outcome was set to .05 before the analysis began, and all reported *P* values are 2-sided. We used R, version 3.3.1 (R Foundation for Statistical Computing) for all statistical analysis.

## Results

### Characteristics of Records Included in Analysis

There were 2580 hospitalizations of patients 18 years or older included on the patient lists from January 1, 2014, to April 30, 2016. On the basis of the eligibility criteria for the program, the following admissions were excluded from analysis: pregnancy related (n = 273), labor and delivery (n = 449), oncology related (n = 83), motor vehicle crash (n = 17), psychiatric only (n = 15), and surgery related (n = 34). We also excluded records for patients who died during their index admission (n = 29), those who were discharged to a short- or long-term care facility (n = 130), or those enrolled in the Camden Coalition’s intensive care management program at the time of their hospital admission (n = 19). Although children were eligible for the 7-DP, our analysis only included adults 18 years or older.

We classified the remaining 1531 patients (mean [SD] age, 48.3 [14.9] years; 888 [58.0%] female; 729 [47.6%] black, non-Hispanic) into treatment and nontreatment groups based on timing of a primary care visit relative to the index discharge date. After the exclusions described above, there were 450 discharges (29.4%) followed by a primary care visit within 7 days, 607 (39.6%) followed up by a primary care visit within 14 days, and 924 (60.4%) with no postdischarge primary care visit within 14 days. The treatment group consisted of 450 discharges among 366 distinct adult patients (mean [SD] age, 48.7 [14.7] years; 289 [64.2%] female; 203 [45.1%] black, non-Hispanic) who attended a primary care visit within 7 days of discharge. The nontreatment pool consisted of 1081 discharges among 772 distinct adult patients (mean [SD] age, 48.1 [14.9] years; 599 [55.4%] female; 526 [48.7%] black, non-Hispanic) with a later primary care visit or no visit. The number of eligible discharges after which a patient attended a postdischarge primary care appointment within 7 days increased from 40 of 207 (19.3%) to 133 of 339 (39.2%) during the study period.

Compared with the nontreatment group, the treatment pool had more female patients (289 [64.2%] vs 599 [55.4%], *P* = .001), Hispanic patients (196 [43.6%] vs 387 [35.8%], *P* = .005), patients with 1 or no mental health comorbidities (370 [82.2%] vs 810 [74.9%], *P* = .001), those with no substance use comorbidities (355 [78.9%] vs 743 [68.7%], *P* < .001), and those with 0 to 4 prior emergency department visits (405 [90.0%] vs 916 [84.7%], *P* = .003) (Table 1).

**Table 1.** Baseline Characteristics of the Full, Unadjusted Cohort by Postdischarge Primary Care Visit Status^a^

Characteristic	No. (%) of Patients			*P* Value
	All	7-d Primary Care Visit		
		Yes (n = 450)	No (n = 1081)	
Age, y^b^				
18-29	234 (15.3)	58 (12.9)	176 (16.3)	.08
≥30	1297 (84.7)	392 (87.1)	905 (83.7)	
Sex				
Male	643 (42.0)	161 (35.8)	482 (44.6)	.001
Female	888 (58.0)	289 (64.2)	599 (55.4)	
Race/ethnicity				
Black, non-Hispanic	729 (47.6)	203 (45.1)	526 (48.7)	.20
White, non-Hispanic	178 (11.6)	43 (9.6)	135 (12.5)	.09
Hispanic	583 (38.1)	196 (43.6)	387 (35.8)	.005
Other	41 (2.7)	8 (1.8)	33 (3.0)	.12
Quan index^c^				
0-3	1242 (81.1)	363 (80.7)	879 (81.3)	.77
≥4	289 (18.9)	87 (19.3)	202 (18.7)	
No. of mental health chronic conditions^c^				
0-1	1180 (77.1)	370 (82.2)	810 (74.9)	.001
≥2	351 (22.9)	80 (17.8)	271 (25.1)	
No. of substance-related chronic conditions^c^				
0	1098 (71.7)	355 (78.9)	743 (68.7)	<.001
1	350 (22.9)	86 (19.1)	264 (24.5)	.02
2	83 (5.4)	9 (2.0)	74 (6.8)	<.001
No. of inpatient admissions in the past 6 mo^d^				
0-1	1188 (77.6)	356 (79.1)	832 (77.0)	.35
≥2	343 (22.4)	94 (20.9)	249 (23.0)	
No. of emergency department visits in the past 6 mo^d^				
0-4	1321 (86.3)	405 (90.0)	916 (84.7)	.003
≥5	210 (13.7)	45 (10.0)	165 (15.3)	

^a^ Records are grouped according to whether a hospitalization was followed by a visit with a primary care provider within 7 days of discharge.

^b^ Patient age at time of discharge.

^c^ The Quan index, mental health chronic conditions grouping, and substance-related chronic conditions grouping were calculated using *International Classification of Diseases, Ninth Revision* and *International Statistical Classification of Diseases and Related Health Problems, Tenth Revision* diagnostic codes in the hospital claims data. Diagnostic codes were grouped according to the clinical classification methods from the Agency for Healthcare Research and Quality.

^d^ Numbers of admissions and emergency department visits in the 6-month period before the index discharge were calculated from hospital claims data.

### Association Between Primary Care Visit Timing and Readmissions

Of the 450 hospitalizations in the treatment group, 57 (12.7%) were followed by any readmission within 30 days vs 78.8 (17.5%) among matched referents (difference, 4.8%; 95% CI, 0.52-9.17; *P* = .03). The mean number of 30-day readmissions was 0.15 (n = 67) for the treatment group compared with 0.22 (n = 100.0) for the nontreatment group (difference, 0.07; 95% CI, 0.02-0.13; *P* = .01). At 90 days after discharge, 126 hospitalizations (28.0%) in the treatment group were followed by any readmission compared with 174 (38.7%) among matched referents (difference, 10.7%; CI, 4.98-16.36; *P* = .002). The mean number of readmissions within 90 days was 0.50 (n = 226) in the treatment group vs 0.63 (n = 283.2) for matched referents (difference, 0.13; 95% CI, 0.01-0.25; *P* = .04). The results based on reweighted records were similar (Table 2).

**Table 2.** Unadjusted, Matched Pairs, and Reweighted Associations Between Postdischarge Primary Care Visit Status and Outcomes

Outcome	7-d Primary Care Visit (n = 450)	Unadjusted Nontreatment Pool (n = 1081)			Matched Pairs Nontreatment Group (n = 450)			Reweighted Nontreatment Group (n = 1081)		
		No 7-d Primary Care Visit	Difference, % (95% CI)^a^	*P* Value	No 7-d Primary Care Visit	Difference, % (95% CI)^a^	*P* Value	No 7-d Primary Care Visit	Difference, % (95% CI)^a^	*P* Value
30-d Readmission rate, No. (%)^b^	57 (12.7)	178 (16.5)	3.80 (0.01-7.58)	.05	78.8 (17.5)	4.84 (0.52-9.17)	.03	192 (17.8)	5.09 (1.27-8.92)	.01
Total readmissions within 30 d, No. (mean)^c^	67 (0.15)	229 (0.21)	0.06 (0.01-0.11)	.01	100.0 (0.22)	0.07 (0.02-0.13)	.01	241.4 (0.22)	0.07 (0.02-0.13)	.005
90-d Readmission rate, No. (%)	126 (28.0)	367 (33.9)	5.95 (0.93-10.97)	.02	174.0 (38.7)	10.67 (4.98-16.36)	.002	375.2 (34.7)	6.71 (1.68-11.73)	.009
Total readmissions within 90 d, No. (mean)	226 (0.50)	683 (0.63)	0.13 (0.02-0.24)	.03	283.2 (0.63)	0.13 (0.01-0.25)	.04	685.8 (0.63)	0.13 (0.01-0.25)	.03

^a^ Differences were calculated as the proportion of records in the nontreatment pool, matched pairs treatment group, or reweighted treatment group with any readmission occurring in 30 or 90 days of index discharge or the mean number of readmissions in 30 or 90 days minus the proportion or mean in the treatment group.

^b^ The rate is the number of records for which the first associated readmission occurred within 30 or 90 days of discharge divided by the total number of records in the appropriate category (treatment or nontreatment).

^c^ The total number of readmissions is the number of readmissions associated with an index discharge that occurred within 30 or 90 days of that discharge. The numerator for the mean is the count of all readmissions for the specified category (treatment or nontreatment); the denominator is the total number of records for that category.

Adding more time for treatment exposure (eTable 2 in the Supplement) or removing records when the patient declined participation or was unresponsive to staff attempts to engage them did not change 30-day readmission outcomes (eTable 3 in the Supplement). Across these models, 30-day readmissions for the treatment group were significantly lower than for the nontreatment group, although the magnitude of the differences varied from the original model. For the model adding more time for treatment exposure, the statistically significant difference in the number of 90-day readmissions between treatment and nontreatment groups held for the reweighted analysis (difference, 0.12; 95% CI, 0.01 to 0.24; *P* = .04) but not for the matched pairs comparison using the mean of 5 records (difference, 0.113; 95% CI, −0.01 to 0.23; *P* = .07).

## Discussion

We observed an association between 7-DP and reduced hospital readmissions for adult Medicaid patients in Camden, New Jersey. At 30 and 90 days after a hospital discharge, patients who attended a primary care appointment within 7 days of discharge had fewer readmissions compared with patients with a later primary care visit or none. These associations persisted after matching patients on demographic and clinical characteristics and prior hospital use.

Like other initiatives associated with reductions in hospital readmissions,^6,15,21^ 7-DP involved intensive efforts to engage patients and support a successful transition from hospital to home. Alongside primary care practices, Camden Coalition staff engaged in continuous quality improvement efforts around practice workflows, bedside engagement, telephone outreach, gift cards and taxis, and reminder calls. Program components included meeting with practices monthly to assess progress, prioritizing relationship building with practice staff at all levels, incentivizing practices to free up timely appointments for patients leaving the hospital, launching promotional campaigns to build momentum for 7-DP, and holding quarterly accountable care organization dinners during which program staff shared results and strategies with providers and other community members.

Although the program’s 7-day appointment rate increased during the study period, less than half of patients had a primary care follow-up appointment within 14 days of a hospital discharge. In Camden, the Medicaid population faces many systemic barriers to primary care access. Even with incentives to ease access, language barriers, lack of social support, competing priorities, unstable housing situations, and mistrust of the health care system were some of the factors that may have impeded program staff’s ability to engage patients and connect them to primary care.

Intensive interventions such as 7-DP can be expensive and inefficient at start-up. However, there are reasons to believe that a reasonable return on investment can be achieved. The program incurred fixed costs and variable costs associated with incentives for successfully completed appointments. Although the study design precluded a more robust analysis of return on investment, a simple analysis of costs and an estimated $10 300 in cost savings per avoided hospitalization suggest that the program would break even if 27 inpatient admissions were avoided annually by connecting 208 patients to primary care within 7 days of hospital discharge (eAppendix 4 in the Supplement).

The return on investment for a program such as 7-DP could be improved through targeted enrollment of patients who would be expected to benefit the most from timely primary care follow-up. The 7-DP program excluded patients who might not benefit from the program based on the reason for their hospital admission, such as surgical patients.^27^ Evidence suggests that patients with the highest medical risk of readmission to the hospital benefit most from timely primary care follow-up,^15,17^ whereas social risk factors may limit the association of timely primary care follow-up with reducing readmission risk.^18^ However, research on the most effective care pathways for high-risk patients after a hospital discharge is inconclusive. An observational study^23^ of readmissions after discharge from a multihospital system found reduced readmissions for high-risk patients with a timely primary care follow-up visit but no reduction among patients at extremely high risk. In a recent study,^28^ researchers concluded that among elderly and chronically ill patients, primary care physician follow-up may contribute more to reducing readmission risk than follow-up with a medical specialist. A critical next step in our evaluation of 7-DP is to identify patient subgroups that benefit most and least from timely primary care follow-up and to recommend programmatic changes based on these findings.

### Strengths and Limitations

Our study has several strengths. To assess hospital utilization before and after an index discharge, we used all-payer claims data linked across 4 hospital systems, improving our ability to capture hospitalizations that might otherwise be missed. In addition, 7-DP removed many noted barriers to primary care visits for patients (eg, transportation) and providers (eg, paying for changes in workflow), plausibly reducing the effect of unmeasured variables that influence primary care follow-up rates. Because program staff recorded when patients declined their help or when staff were unable to contact a patient, we were able to account for some unmeasured differences in the patient population by excluding these patients from the analysis and comparing results.

Our study also has limitations. The benefits that we observed for a timely primary care follow-up after hospital discharge may still reflect unmeasured differences in the patient population and the quality of care received during a primary care visit. Although we conducted a sensitivity analysis that excluded patients who declined to participate or who were unreachable, other unmeasured variables may have influenced our results. For example, the strength and quality of the relationship between patients and providers might influence who has a timely follow-up appointment and who is more or less likely to be readmitted to the hospital. Social risk variables, such as housing and food security, that could affect health services utilization were also not included in our data. Consistency of Medicaid coverage over time could also influence readmission patterns. Furthermore, because we did not have comparable data about primary care connection rates before the program, we are unable to draw conclusions about the association of the program with improvement in primary care follow-up rates or reduction of readmissions. Also, data quality issues are common across the data sources we used. We completed multiple validations of the data sets to ensure that data quality issues did not overly influence our results, but errors could remain.

## Conclusions

Timely connection to primary care after a hospital discharge creates opportunities to discuss medication changes and other discharge instructions outside the hospital setting, potentially reducing readmission risk. Programs such as the 7-DP may be associated with a reduction in preventable hospital admissions through patient and practice engagement, providing incentives to patients to overcome barriers to keeping an appointment, and adequately reimbursing practices on top of regular reimbursement and value-based payments to prioritize appointments for recently discharged patients. Program return on investment could be improved by targeting patients for whom timely primary care follow-up would be expected to have the largest association with reducing readmission risk.

## Notes

Supplement. **eAppendix 1.** Data Linkage **eAppendix 2.** Logistic Regression Modeling to Produce Propensity Scores **eAppendix 3.** Standard Error Calculation for Nearest Neighbor Propensity Score Matching **eAppendix 4.** Return on Investment Calculation **eTable 1.** Propensity Score Balance of Each Covariate Before and After Matching **eTable 2.** Readmission Results for Sensitivity Analysis Shifting Baseline for Counting Readmissions From Day 1 After Discharge to Day 8 After Discharge **eTable 3.** Readmission Results for Sensitivity Analysis Excluding Patients Who Refused Services or Were Unreachable in the Hospital or Following Discharge From the Hospital **eFigure.** Distribution of Propensity Scores for Treatment Group and Non-Treatment Pool **eReferences**
