# Supplementary material for: Outcomes of a Citywide Campaign to Reduce Medicaid Hospital Readmissions With Connection to Primary Care Within 7 Days of Hospital Discharge
Source: JAMA Netw Open. 2019 Jan 25;2(1):e187369. doi: 10.1001/jamanetworkopen.2018.7369 (PMC6484580; doi:10.1001/jamanetworkopen.2018.7369)
Supplement: Supplement. — eAppendix 1. Data Linkage eAppendix 2. Logistic Regression Modeling to Produce Propensity Scores eAppendix 3. Standard Error Calculation for Nearest Neighbor Propensity Score Matching eAppendix 4. Return on Investment Calculation eTable 1. Propensity Score Balance of Each Covariate Before and After Matching eTable 2. Readmission Results for Sensitivity Analysis Shifting Baseline for Counting Readmissions From Day 1 After Discharge to Day 8 After Discharge eTable 3. Readmission Results for Sensitivity Analysis Excluding Patients Who Refused Services or Were Unreachable in the Hospital or Following Discharge From the Hospital eFigure. Distribution of Propensity Scores for Treatment Group and Non-Treatment Pool eReferences [file jamanetwopen-2-e187369-s001.pdf]

Supplementary Online Content

Wiest D, Yang Q, Wilson, C, Dravid N. Outcomes of a citywide campaign to reduce Medicaid hospital readmissions with connection to primary care within 7 days of hospital discharge. *JAMA Netw Open.* 2019;2(1):e187369. doi:10.1001/jamanetworkopen.2018.7369

eAppendix 1. Data Linkage

eAppendix 2. Logistic Regression Modeling to Produce Propensity Scores

eAppendix 3. Standard Error Calculation for Nearest Neighbor Propensity Score Matching

eAppendix 4. Return on Investment Calculation

eTable 1. Propensity Score Balance of Each Covariate Before and After Matching

eTable 2. Readmission Results for Sensitivity Analysis Shifting Baseline for Counting Readmissions From Day 1 After Discharge to Day 8 After Discharge

eTable 3. Readmission Results for Sensitivity Analysis Excluding Patients Who Refused Services or Were Unreachable in the Hospital or Following Discharge From the Hospital

eFigure. Distribution of Propensity Scores for Treatment Group and Non-Treatment Pool

eReferences

This supplementary material has been provided by the authors to give readers additional information about their work.

© 2019 Wiest D et al. *JAMA Network Open.*

eAppendix 1. Data linkage

Data sets

Data used in this analysis include internal programmatic data that record staff interactions with patients, patient insurance eligibility information provided by payers, Camden Health Information Exchange records, and all-payer claims data from four regional health care systems. Administrative records from these sources were linked to obtain a more complete picture of patients' medical encounters across all institutions. Each data set is described below.

A) Medicaid managed care organization (MCO) eligibility files

Two Medicaid MCOs, UnitedHealthcare and Horizon NJ Health, provided monthly lists of their eligible members to the Camden Coalition of Healthcare Providers (Camden Coalition). Any individuals who appeared on these lists between January 2014 and April 2016 were included for the purposes of the data file linkage. The MCO files included 86727 records with 77194 unique combinations of gender, date of birth, first name, and last name.

Fields from the MCO eligibility files that were used in linkage:

Field	Description
subscriber_id	MCO-generated account id, nominally unique per person within a given MCO's records. In practice, individuals can have more than one id over time.
ssn	Beneficiary Social Security Number
gender	Beneficiary gender (binary)
last_name	Beneficiary last name
first_name	Beneficiary first name
dob	Beneficiary date of birth
hieid	An internally generated id number indicating that the given patient was able to be matched to records in the Camden Health Information Exchange.

© 2019 Wiest D et al. *JAMA Network Open.*

B) All-payer hospital claims data

The Camden Coalition has a dataset of hospital insurance claims from 2010 through 2016 containing 6899261 records from all inpatient, emergency, observation encounters and a limited number of outpatient encounters from regional health care systems at several sites. All-payer claims from the following health care systems are included in the database: Cooper University Health Care, Lourdes Health System, Virtua Health System, and Kennedy Health System (now Jefferson Health).

Fields from hospital claims data that were used in linkage:

Field	Description
link_id	The unique identifier for a given set of records previously linked together and thus assumed to belong to the same real patient
ssn	Patient Social Security Number
gender	Hospital-recorded patient gender
last_name1	Patient last name
last_name2	Second part of a two-part hyphenated name
first_name	Patient first name
dob	Date of birth
visit_type	The visit type: inpatient, emergency department, observational, outpatient

C) Camden Health Information Exchange

The Camden Coalition's 7-Day Pledge daily workflow began with triaging inpatient records from the Camden Health Information Exchange (CHIE) to identify patients eligible for the intervention.

© 2019 Wiest D et al. *JAMA Network Open.*

Field	Description
ssn	Patient Social Security Number
gender	Patient Gender
last_name	Patient last name
first_name	Patient first name
dob	Patient date of birth
AdmitDate	The admission date of the hospitalization or date seen in the emergency department
DischargeDate	The discharge date of the hospitalization
Visit_type	The visit type: inpatient or emergency department

Fields from the Camden Health Information Exchange that were used in linkage:

Data Cleaning

Several cleaning steps were performed on the data files before they were linked. Two or more records with identical values in all but one field were combined into a single record. Because there were no facially invalid genders or birth dates, no values for these variables were removed before linkage.

Social security numbers

After removing all non-numerals and anything with more than or less than 9 digits, Social Security Numbers (SSNs) with impermissible, or overly common default values - even where the latter otherwise follow a valid pattern - were removed because they are not unique to an individual and thus would be more likely to cause records to be improperly linked together. Removed SSN's included those ending in 4 identical digits, having a sequence of 5 or more consecutive identical digits, starting with 666 or 999, or consisting of only 2 different digits regardless of the pattern. Some sequences of numbers occurring in particular positions are never used by the Social Security Administration (SSA). These were removed, but care was taken to leave in the number patterns that are not used in "true" SSNs but which are used in non-citizen tax ID numbers or temporary numbers for those awaiting an SSN. There are also several SSNs that have been invalidated by the Social Security Administration because they have been used as examples in advertising, instructions on common documents, or other similar situations.

© 2019 Wiest D et al. *JAMA Network Open.*

In addition, there were some SSN anomalies specific to the data files that were also removed. Some SSN values were actually composed of 3 random or repeated digits followed by 6 digits in sequence from the date of birth.

Names

First names that were generic values indicating a newborn who had not yet been named at the time the record was created were removed. Name suffixes (e.g. “Sr.”), prefixes (e.g. “Mr.”), and middle initials were parsed out into separate fields since they are inconsistently captured in administrative data, adding a false specificity. Names with internal spaces (“San Gabriel”, “Von Trapp”, “Jo Ann” etc.) had those spaces collapsed for the same reason. Hyphenated surnames were split into “last_name1” and “last_name2”. Where possible, surnames that would have been hyphenated but which had been compressed into a single name without hyphens or spaces were also split into two last name fields by comparing them to a list of known two-part names. Administrative name capture is often not well adapted to multipart surnames such as many Hispanic names, which are often recorded or provided in different order or using only one of the two parts in different times and settings. Records with artificial name values like “SEE MEMO” had those values removed before linkage.

Data linkage methodology MCO↔Claims

Each record with the same hieid *or* subscriber id *or* cleaned ssn *or* the same gender, date of birth, phonetic first_name, *and* phonetic last_name1 *or* the same gender, date of birth, phonetic first_name, *and* phonetic last_name2 was considered to belong to the same individual. When a record belonged to more than one grouping the groups with a record or records in common were merged. This transitive linkage process was repeated until all remaining groups did not overlap.

A phonetic transformation of a name substitutes standardized representations of phonemes for letter sequences in a way that emphasizes most consonants over most vowels subject to other general pronunciation rules. The phonetic transformation serves two purposes: 1) it accounts for many transcription errors from spoken information; and 2) it generalizes the names to minimize the noise introduced by many types of spelling and typographic errors. The phonetic algorithm used was “double metaphone” rather than the more common SOUNDEX because it is intended to be more sensitive to a wider variety of pronunciations.[1-2]

The linkage is conservative about dates of birth and gender, more flexible with names, and literal but trusting about SSN, allowing it to be sufficient on its own to link records. Because SSN can therefore have an outsized impact on linkage, the results were reviewed to screen out any more extreme cases of false-positive over-linking that may have occurred. Eight nominally valid SSNs occurred multiple times in the dataset with an array of entirely different names, subscriber ids, dates of birth, and genders in a way that caused the formation of large groups of overlapping but clearly unrelated records. These SSNs were removed and the linkage process was repeated.

© 2019 Wiest D et al. *JAMA Network Open.*

As a final step, the linked records were compared on SSN, gender, birthdate, and name combinations as described above to the previously linked claims records so that any groups of eligibility records that matched to the same linked group in the claims data or any one eligibility group that was matched to more than one claims linked group (total n = 306) was inspected for possible false positive links and split apart if needed based on clerical review.

Data linkage methodology (MCO↔Claims)↔CHIE

After the linkage between MCO and Claims data sets was complete, we obtained a dataset consisting of hospital encounters for the population listed in the MCO Eligibility Files. We then linked that data to data in the CHIE. The goal was to verify that each record in the client tracking system did exist in the hospital claims data. We performed a strict deterministic linkage at this stage with the following matching conditions:

1. Subscriber_ID, last name, first name, date of birth, and gender should be matched;
2. Visit type must be inpatient;
3. Admit date and discharge date should be matched exactly.

After this linkage, we found that around 90% of our records could be found exactly in the hospital claims data. For the unmatched 10%, we further diagnosed and grouped them into three categories:

1. Patients' identifiers matched, admit dates and discharge dates matched, but visit types were not matched;
2. Patients' identifiers matched, visit types matched, but either admit date or discharge date were not matched;
3. Patients' identifiers matched, but none of the visit types, admit dates, or discharge dates matched.

Our care team staff further looked into the electronic medical records to diagnose situations 1 and 2 above. We found that for visit type, it was difficult to determine which data source was more reliable since some dates were correct in the hospital claims data and some were correct in the CHIE. For the dates mismatched in 2, we found that hospital claims data were more reliable, and therefore updated the dates based on the dates from the hospital claims data. For situation 3, we applied a "3-day rule," which meant that if we found an inpatient record from the hospital claims data for the same patient that differed by only three days from our CHIE dates, we believed they were the same records and we used the dates from the hospital claims data.

eAppendix 2. Logistic regression modeling to produce propensity scores

We used logistic regression to develop the propensity scores for our analysis. The table below lists the eight covariates included in the model, their source, variable type, and the range of values before they were transformed.

Covariates included in logistic regression model

Covariate	Data source	Variable type	Raw values
Age at discharge	Discharge Date: claims data<br><br>DOB: MCO capitation list (validated using claims data)	Scale	18.0- 92.9
Gender	MCO capitation list (validated using claims data)	Nominal	Female, Male
Race & Ethnicity	Claims data	Nominal	Black, non-Hispanic; White, non-Hispanic; Hispanic; Other
Quan index	Claims data	Scale	0-22
No. mental health chronic conditions	Claims data	Scale	0-7
No. substance use chronic conditions	Claims data	Scale	0-2
No. of inpatient admissions 6 months prior to index discharge	Claims data	Scale	0-15
No. of emergency department visits 6 months prior to index discharge	Claims data	Scale	0-50

© 2019 Wiest D et al. *JAMA Network Open.*

The Quan index, used to capture the count and severity of medical chronic conditions, and mental health and substance use chronic conditions, was calculated using the ICD9 and ICD10 diagnostic codes in the claims data. We grouped the diagnostic codes based on the clinical classification methods from the Agency for Healthcare Research and Quality (AHRQ).[3-6] Each group category was counted at most once. We used the R package ICD to compute the Quan index.

We performed bivariate analysis to determine the form of each covariate to include in our model. Decisions were based on empirical distributions and each variable's association with post-discharge primary care appointment timing, the dependent variable. In some cases (e.g. number of emergency department visits and number of chronic mental health conditions) we grouped the data to smooth the extreme effect of outliers. We maintained the original distribution for the number of substance use diagnoses because of the large differences in the 7-day primary care appointment rate for patients with 0, 1, and 2 diagnoses: 355 out 1098 (32%) of patients with 0 substance use diagnoses had a primary care appointment within 7 days of discharge, compared to 86 of 350 (25%) for patients with 1 substance use chronic condition, and 9 of 83 (11%) patients with 2 substance-related chronic conditions.

To build our full model and generate propensity scores, we used forward selection based on the Akaike Information Criterion (AIC).[7] The details of our logistic regression model and a comparison of the fit of the model with the raw values to the fit of the model with the transformed values are shown in the table below. Propensity scores were produced from the model with the transformed covariates. Although age, Quan index, and prior hospitalizations showed no significant relationship to the timing of a post-discharge primary care appointment, they were included in the final model to produce the propensity scores because of their clinical relevance.

© 2019 Wiest D et al. *JAMA Network Open.*

Binary logistic regression results examining the relationship between timing of post-discharge primary care follow-up and covariates in raw and transformed format (n=1531)[a]

Model 1: Covariates in raw format				Model 2: Covariates in transformed format			
Covariate	Coefficient	S.E.	P-value	Covariate[b]	Coefficient	S.E.	P-value
(Intercept)	-0.732	0.221	0.001	(Intercept)	-0.532	0.112	0.000
Age	0.003	0.004	0.38	Age (18—30)	-0.376	0.170	0.03
Gender	-0.325	0.120	0.007	Gender (Male)	-0.332	0.120	0.006
Quan index	-0.009	0.021	0.69	Quan index (4+)	-0.035	0.150	0.81
No. of mental health chronic conditions	-0.088	0.060	0.15	No. of mental health chronic conditions (2+)	-0.331	0.149	0.03
No. of substance use chronic conditions	-0.399	0.118	0.001	No. of substance use chronic conditions	-0.381	0.116	0.001
No. of inpatient admissions 6 months prior to index discharge	0.043	0.034	0.21	No. of inpatient admissions 6 months prior to index discharge (2+)	0.052	0.149	0.73
No. of emergency department visits 6 months prior to index discharge	-0.028	0.017	0.09	No. of emergency department visits 6 months prior to index discharge (5+)	-0.344	0.193	0.07
Race & Ethnicity: Hispanic	0.208	0.124	0.09	Race & Ethnicity: Hispanic	0.247	0.124	0.04
Race & Ethnicity: Other	-0.614	0.406	0.13	Race & Ethnicity: Other	-0.563	0.407	0.17
Race & Ethnicity: White, non-Hispanic	-0.187	0.199	0.35	Race & Ethnicity: White, non-Hispanic	-0.166	0.199	0.40
Drop in 2(log Likelihood full model – log Likelihood null model)	23	NA	0.000		26.29	NA	0.000
Percentage of cases that the full model classifies correctly	67.28%	NA			72.96%	NA	
Drop in Akaike Information Criterion (AIC)[c]	25.4	NA			32.5	NA	

a. Models include 450 “treatment” records and 1081 “non-treatment” records.
b. See table above for reference values.
c. The absolute difference in the AIC value for the model with the raw values of the covariates and the model with the transformed values (|25.4-32.5|=7.1) suggests there is “considerably less support” for the former compared to the latter.[8-9]

© 2019 Wiest D et al. *JAMA Network Open.*

eAppendix 3. Standard error calculation for nearest neighbor propensity score matching

For each patient in the treatment group, we used nearest neighbor matching with replacement to identify five matched referents in the non-treatment pool. To quantify uncertainty, we used a method for calculating standard error for nearest neighbor matching without replacement discussed in the work of Michael Lechner.[10-11] The formula is as follows:

$$\boldsymbol{Var(average\ treatment\ effect\ on\ the\ treated\ (ATT)) =}$$
$$\frac{\mathbf{1}}{\boldsymbol{N1}}\boldsymbol{Var(measure\ on\ the\ treatment\ group)} + \frac{\sum_j \boldsymbol{w_j}^2}{(\boldsymbol{aN1})^2} * \boldsymbol{Var(measure\ on\ the\ matched\ sample)}$$

Where *N1* is the number of matched treated individuals, *w_j* is the number of times individual *j* from the control group has been used, and *a* is the number of nearest neighbors that we select for each treatment record. Lechner found little difference between bootstrapped variances and the variances calculated according to the above equation.[10-11]

© 2019 Wiest D et al. *JAMA Network Open.*

eAppendix 4. Return on investment calculation

To estimate return on investment for the 7-DP program, we considered fixed costs and variable costs, along with the estimated savings associated with avoided hospitalizations.

- Fixed costs to run the program for a year were $230000.
- The variable costs associated with each successful 7-day primary care visit were $200, made up of a $150 incentive payment, a $22.50 gift card for the patient (includes fees not passed on to the patient for a $20 gift card), and a round trip taxi which we estimate at $27.50. Taxi fare is a conservative estimate, as only about 25% of patients required a taxi and many rides were cheaper than this estimate.
- Based on the analysis of claims data during the period evaluated, we estimated that each avoided hospitalization created an average of $10300 in cost savings.

Using these figures, we applied the following reasoning to determine the number of patients needed to treat in a year for the program to break even:

- Based on the analysis presented in the paper, the treatment 90-day average number of admissions was 0.502 and the non-treatment 90-day average was 0.629 for a difference of .127 admissions. Each successful visit was associated with a 0.127 inpatient reduction within a 90 day period.
- For **X** 7-day visits, 0.127***X** inpatient admissions will be avoided.
- Total annual costs= fixed costs ($230000) + variable costs ($200***X**).
- Estimated cost per avoided hospital admission is $10300.
- Total estimated cost savings= 0.127***X***$10300.
- Number of visits needed to break even: $230000+200**X**=0.127***X***$10300.
- **X**=208 visits.
- The number of inpatient reductions will be: 0.127*208=26.4=27.
- Our 7-day appointment success rate averaged 30% over the study period.
- If we assume 30% 7-day appointment success rate per year, to achieve 208 successful visits, we will need 208/0.3=694 attempted engagements.
- 694 engagements per year works out to just under 3 patients per day based on 20 work days in a month. This is a manageable benchmark given the same number and level of staff around which the fixed costs were estimated.

© 2019 Wiest D et al. *JAMA Network Open.*

eTable 1. Propensity score balance of each covariate before and after matching

Covariates	Treatment group (n=450)	Non-treatment group before matching (n=1081)	Non-treatment group after matching (n=450)	Standardized bias after matching
Age: 18-30	12.89%	16.28%	11.82%	0.03
Gender: Male	35.78%	44.59%	38.62%	0.06
Race: Black, non-Hispanic	45.11%	48.66%	45.69%	0.01
Race: White, non-Hispanic	9.56%	12.49%	9.69%	0.005
Race: Hispanic	43.56%	35.80%	43.64%	0.002
Race: Other	1.78%	3.05%	0.98%	0.06
Quan Index: High	19.33%	18.69%	17.51%	0.05
Substance-related conditions: 0	78.89%	68.73%	79.56%	0.02
Substance-related conditions: 1	19.11%	24.42%	18.53%	0.01
Substance-related conditions: 2	2.0%	6.85%	1.91%	0.002
Mental health conditions: 2+	17.78%	25.07%	19.29%	0.04
Prior 6 month ED use: 5+	10.00%	15.26%	9.11%	0.03
Past 6 month admissions: 2+	20.89%	23.03%	19.56%	0.03

a. If the covariate is a binary variable, only one level is shown above. The excluded level will be 1 minus the percentage shown in table.

b. Standardized bias was calculated as the difference in proportions of each level of the measured covariate divided by the standard deviation in the treatment group.[12,13]

© 2019 Wiest D et al. *JAMA Network Open.*

eTable 2. Readmission results for sensitivity analysis shifting baseline for counting readmissions from day 1 after discharge to day 8 after discharge

Outcome	7-day Primary Care Visit (n= 450)	Unadjusted Non-treatment pool (n= 1081)			Matched Pairs Non-treatment group (n= 450)			Reweighted Non-treatment group (n= 1081)		
		No 7-day Primary Care Visit	Difference (95% CI)[c]	p-value	No 7-day Primary Care Visit	Difference (95% CI)	p-value	No 7-day Primary Care Visit	Difference (95% CI)	p-value
30-day readmission rate, No. (%)[a]	68 (15.1)	201 (18.6)	(-0.59, 7.52)	0.09	100.4 (22.3)	(2.51, 11.89)	0.003	213.6 (19.8)	(0.58, 8.73)	0.03
Total readmissions within 30 days, No. (Mean)[b]	78 (0.17)	252 (0.23)	(0.01, 0.11)	0.02	121.6 (0.27)	(0.04, 0.16)	0.002	263.1 (0.24)	(0.02, 0.12)	0.01
90-day readmission rate, No. (%)	134 (29.8)	376 (34.8)	(-0.00, 10.09)	0.05	175.6 (39.0)	(3.49, 14.99)	0.002	385.7 (35.7)	(0.80, 10.99)	0.02
Total readmissions within 90 days, No. (Mean)	234 (0.52)	692 (0.64)	(0.01, 0.23)	0.04	284.8 (0.63)	(-0.01, 0.23)	0.07	696.3 (0.64)	(0.01, 0.24)	0.04

a. The rate is the number of records for which the first associated readmission occurred within 30 or 90 days of discharge divided by the total number of records in the appropriate category (treatment or non-treatment).
b. The total number of readmissions is the number of readmissions associated with an index discharge that occurred within 30 or 90 days of that discharge. The numerator for the mean is the count of all readmissions for the specified category (treatment or non-treatment); the denominator is the total number of records for that category.
c. Differences were calculated as the proportion of records in the non-treatment pool, matched pairs treatment group, or reweighted treatment groups with any readmission occurring in 30 or 90 days of index discharge, or the mean number of readmissions in 30 or 90 days, minus the proportion or mean in the treatment group.

© 2019 Wiest D et al. *JAMA Network Open.*

eTable 3. Readmission results for sensitivity analysis excluding patients who refused services or were unreachable in the hospital or following discharge from the hospital[a]

Outcome	7-day Primary Care Visit (n= 437)	Unadjusted Non-treatment pool (n=823)			Matched Pairs Non-treatment group (n=437)			Reweighted Non-treatment group (n=823)		
		No 7-day Primary Care Visit	Difference (95% CI)[d]	p-value	No 7-day Primary Care Visit	Difference (95% CI)	p-value	No 7-day Primary Care Visit	Difference (95% CI)	p-value
30-day readmission rate, No. (%)[b]	56 (12.8)	145 (17.6)	(0.73, 8.88)	0.02	103.8 (23.8)	(6.30, 15.58)	0.001	150.9 (18.3)	(1.42, 9.62)	0 .01
Total readmissions within 30 days, No. (Mean)[c]	66 (0.15)	185 (0.22)	(0.02, 0.13)	0.008	121.8 (0.28)	(0.07, 0.19)	0.001	189.1 (0.23)	(0.02, 0.13)	0.008
90-day readmission rate, No. (%)	123 (28.2)	293 (35.6)	(2.12, 12.79)	0.006	202.2 (46.3)	(12.31, 23.94)	0.001	295.4 (35.9)	(2.40, 13.08)	0.004
Total readmissions within 90 days, No. (Mean)	223 (0.51)	541 (0.66)	(0.02, 0.27)	0.02	311.0 (0.71)	(0.08, 0.32)	0.001	539.8 (0.66)	(0.02, 0.27)	0.02

a. This analysis excludes 52 cases for which the patient refused to schedule an appointment through the program and 219 cases for which program staff were unable to reach the patient in the hospital or after discharge. If the patient was unreachable in the hospital, program staff attempted to reach the patient for up to 7 days following discharge by phone or through a letter sent to the patient's home address.
b. The rate is the number of records for which the first associated readmission occurred within 30 or 90 days of discharge divided by the total number of records in the appropriate category (treatment or non-treatment).
c. The total number of readmissions is the number of readmissions associated with an index discharge that occurred within 30 or 90 days of that discharge. The numerator for the mean is the count of all readmissions for the specified category (treatment or non-treatment); the denominator is the total number of records for that category.
d. Differences were calculated as the proportion of records in the non-treatment pool, matched pairs treatment group, or reweighted treatment groups with any readmission occurring in 30 or 90 days of index discharge, or the mean number of readmissions in 30 or 90 days, minus the proportion or mean in the treatment group.

© 2019 Wiest D et al. *JAMA Network Open.*

eFigure. Distribution of propensity scores for treatment group and non-treatment pool

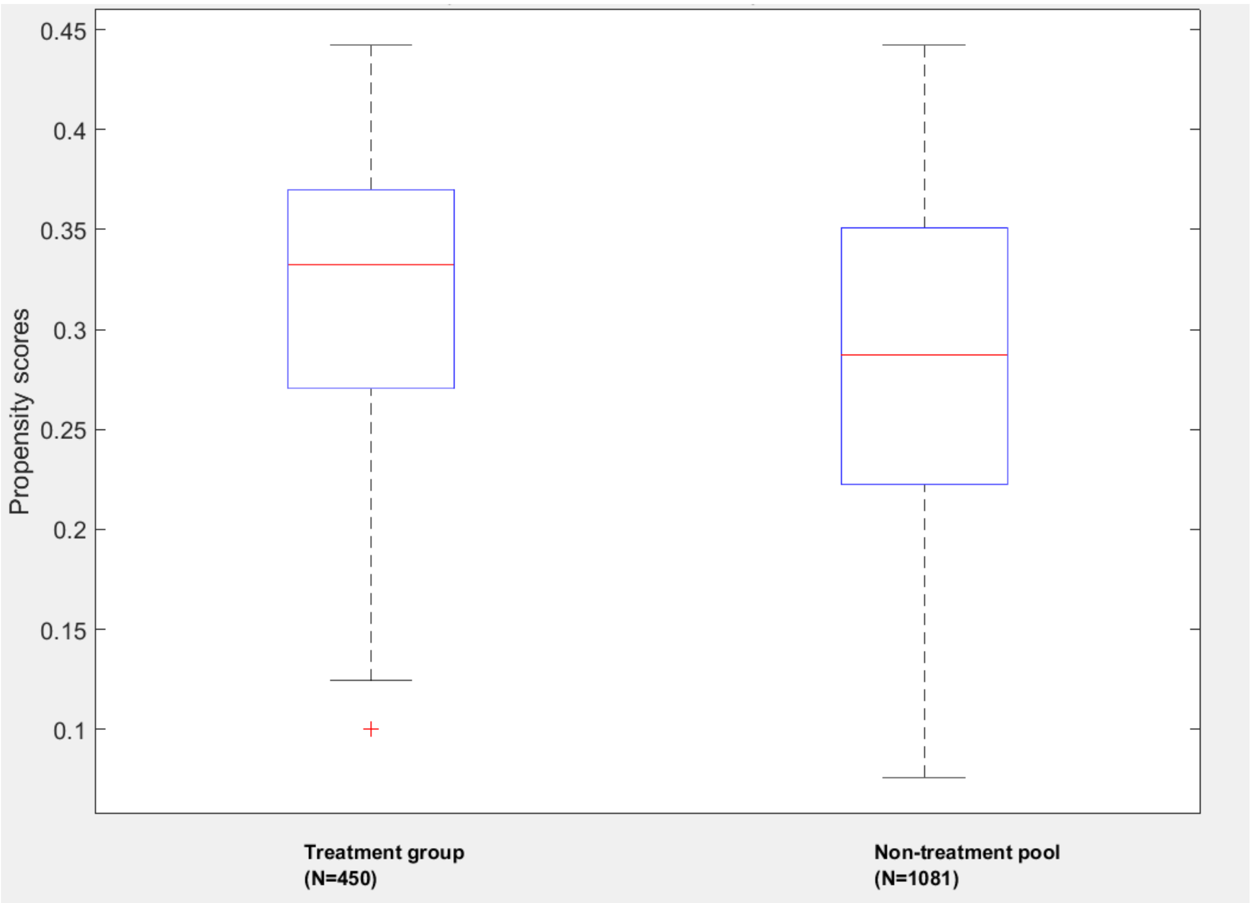

© 2019 Wiest D et al. *JAMA Network Open.*

eReferences

1. Philips, L. The double metaphone search algorithm. *C/C++ Users Journal*. 2000;18(6):38-43.

2. Patman F, Shaefer L; Language Analysis Systems, Inc. Is soundex good enough for you? On the hidden risks of Soundex-based name searching. https://www.immagic.com/eLibrary/ARCHIVES/GENERAL/LAS_US/L030206B.pdf. Published 2001. Accessed September 5, 2018.

3. *Clinical classifications software (CCS) for ICD-9-CM* [source code]. Rockville, MD: Agency for Healthcare Research and Quality, Healthcare Cost and Utilization Project; 2017.

4. *Beta clinical classifications software (CCS) for ICD-10-CM/PCS* [source code]. Rockville, MD: Agency for Healthcare Research and Quality, Healthcare Cost and Utilization Project; 2018.

5. *HCUP chronic condition indicator* [source code]. Rockville, MD: Agency for Healthcare Research and Quality, Healthcare Cost and Utilization Project; 2016.

6. *Beta chronic condition indicator (CCI) for ICD-10-CM* [source code]. Rockville, MD: Agency for Healthcare Research and Quality, Healthcare Cost and Utilization Project; 2018.

7. Akaike H. Information theory and an extension of the maximum likelihood principle. In: Petrov BN and Csaki F, eds. *Proceedings of the 2nd International Symposium on Information Theory*. Budapest, Hungary: Akademiai Kiado; 1973:267-281.

8. Burnham KP, Anderson DR. *Model selection and multimodel inference: a practical information-theoretic approach*. New York, NY: Springer Verlag New York; 2002.

9. Petrossian GA, Maxfield M. An information theory approach to hypothesis testing in criminological research. *Crime Science*. 2018; 7(1):2.

10. Lechner M. Identification and estimation of causal effects of multiple treatments under the conditional independence assumption. In: Lechner M, Pfeiffer F, eds. *Econometric Evaluation of Labour Market Policies*, Heidelberg, Germany: Physica; 2001:43-58.

11. Lechner M. Some practical issues in the evaluation of heterogeneous labor market programmes by matching methods. *J R Stat Soc Ser A Stat Soc*. 2002;165(1):59-82.

12. Rosenbaum PR, Rubin DB. Constructing a control group using multivariate matched sampling methods that incorporate the propensity score. *Am Stat*. 1985b; 39:33-38.

13. Austin P. Using the standardized difference to compare the prevalence of a binary variable between two groups in observational research. *Commun Stat Simul Comput*. 2009; 38(6):1228–1234.

© 2019 Wiest D et al. *JAMA Network Open.*
